# Notes and Observations in General Practice

**Published:** 1868-08-01

**Authors:** John Forman

**Affiliations:** Edinburgh


### NOTES AND OBSERVATIONS IN GENERAL PRACTICE.

BY JOHN FORMAN, M.D., EDINBURGH.

### Paper I.—Acupressure.

Acupressure, first proposed by Sir James Simpson, of Edinburgh, may be truly termed a great acquisition, deserving the attention of every surgeon in the land.

It has been proposed in Edinburgh, also in the Aberdeen Infirmary, by Professor Pirrie and Dr. Keith, to be at once as reliable a hæmostatic agent as the ligature, possessing the advantage of much easier application, no assistant being required as in the case of the ligature. But its great superiority is the acquisition of union by the first intention, or by primary adhesion without the formation of pus. In the case of the ligature we are all aware that such a happy result can not be obtained, the laceration of two of the coats of the artery, the strangulation and destruction of the external coat, the ligature running for several days as a seton in the wound, under suppuration more or less inevitable, consequently heading by the first intention can not be obtained. We are, therefore, warranted in urging the great superiority of acupressure over the ligature as a reliable and expeditious hæmostatic agent with the additional and most important advantage of promoting healing by first intention.

Notwithstanding a certain amount of lukewarmness (and, in some quarters, strong opposition), which every surgical discovery has met with more or less in its infancy, acupressure has already obtained the confidence of, and is practiced by many British surgeons of eminence, and its youthful vigor bids fair to lead us to anticipate, that ere long it will become the exclusive method for arresting hæmorrhage in the medium and larger sized arteries, torsion being quite as effective, and more simple and expeditious for the smaller ones.

I will briefly state the various modes proposed by Professor Simpson.

The first method is performed by passing the needle from the cutaneous surface, directly through the whole thickness of the flaps, and causing it to emerge a little to the right side of the tube of the vessel. The projecting end is then pressed firmly against the site of the artery, made to rëenter the flaps close to the right side of the vessel, and pressed until it emerges on the surface of the skin. In this method, the artery is compressed against the component parts of the flap.

The second method is performed with a needle threaded with twisted iron wire, and, unlike as in the first method, the skin is not interfered with; the needle is passed above instead of below the artery. The needle is pushed twice into the soft tissue of the wound. The first point of entrance is at a little distance from the artery to be acupressed, and the first point of exit close to it. The second point of entrance is close to the vessel on its opposite side, and the second point of exit at a little distance. Between the first point of exit and the second point of entrance, the needle is made to bridge over the trunk of the artery, and care must be taken before making the needle rëenter the wound to pass down sufficiently to close the artery. The needle can be removed at pleasure by pulling the twisted wire.

The third method requires for its performance a threaded needle, and a loop of inelastic wire, and consists in effecting compression between the needle below, and the loop above the vessel. The needle is entered a few lines to one side of the vessel, and pushed behind it, caused to emerge a few lines beyond the vessel; the loop of wire is thrown over the point; brought over the trunk of the artery, and behind the stem of the eye-end of the needle, drawn sufficiently to shut the vessel, and fixed by a half twist around the needle. It is important in the performance of this method, to avoid including an unnecessary amount of tissue ; not to draw the wire tighter than is absolutely necessary to close the artery. By pulling the twisted wire the needle is removed, and the loop being liberated, is easily withdrawn.

The fourth method differs from the third, inasmuch as a long pin is substituted for the threaded needle. Little as the difference may appear, the pin should be substituted in all cases where the form of the wound, and the position of the artery admit of the head of a pin being conveniently, and without straining of tissue, kept without the wound. The pin is easier inserted and withdrawn.

There are several other methods adopted by various surgeons. Such as the twist and ring, adopted by Professor Pirrie and Dr. Keith, of Aberdeen ; and others will, no doubt, in time suggest themselves. In the meantime, the third and fourth methods are the most satisfactory and efficient means of arresting hæmorrhages we possess.

According to the magnitude of the operation in which acupressure has been adopted, the needles or pins are withdrawn in from four to forty-eight hours after its performance.

It is believed by those who have adopted this method in a large number of cases, that less than four hours will be necessary in those operations.

In the minor walks of surgery, such as wounds over the temple, the introduction of a needle under the temporal artery, and a thread passed over it in the form of a figure 8, from eye to point, and secured, is the most efficient mode of arresting the hæmorrhage. In the same manner the needle may be passed under the facial artery as it crosses the lower jaw to arrest troublesome bleeding from deep gashes in the cheek ; and in cases of wounds of the face, where it is important that little or no mark should be left, a fine needle, passed obliquely from one edge of the wound to the other, and a thread passed over it in the manner already described, when heating by first intention invariably ensues, and little or no mark remains.

In cases of deep laceration and severe bruising of the face from falls or blows, on removal of the needle, the thread may be left to form a scab, and painted over daily with a weak solution of carbolic acid and glycerine. In such cases, where much bruising has been sustained, more or less suppuration is unavoidable. This is, however, very much lessened by adopting the carbolic acid dressing, either in conjunction with glycerine, or in the form of paste, as recommended by Professor Lester, of Glasgow, which seems to destroy most effectually the germs, whether they are generated in the atmosphere or in hospital wards, and very materially curtails pus formation, and accelerates the healing process.

In scalp wounds our colleges have hitherto taught us to treat them as follows, viz.: To remove the hair with scissors, a considerable portion of the scalp to be shaved to allow plaster to adhere—the clots of blood to be removed, the wound to be well washed, and should bleeding be troublesome, torsion of some of the vessels should be resorted to. When the edges are brought together, adhesive plaster and a cold compress are to be applied. A third injunction is laid down, not to use ligatures or sutures of wire, lest erysipelas should intervene. Contrast this with the following simple, expeditious and highly satisfactory method. In such a case the clots are removed, the wound thoroughly washed, a pin a little longer than the extent of the wound is inserted through the skin at the lower angle of, and about a quarter of an inch from the edge of the wound. The pin is then run along the raw surface of the lip, till the upper angle is reached, when the point of the pin is made to appear through the skin at the proper level and distance from the raw edge; then a few turns of ordinary linen thread are passed in figure 8 form, from head to point of the pin. All bleeding vessels are thus completely compressed between the pin against the tissues below, and the thread above. A second pin is applied in a similar way along the other lips of the wound, and thread applied over it, same as in the first. The edges of the wounds are brought together, and kept in close apposition by a few turns of thread passed from the head of one pin to the point of the other, and *vice versa*. The hair is then brought over the wound, and the dressing is complete. The pins may be withdrawn in from twenty-four to forty-eight hours, and the thread allowed to remain until healing by first intention is completed, and no fear of erysipelas or other untoward symptom need be anticipated.

PAPER II.—RADICAL TREATMENT OF CHRONIC BLENNORRHŒA.

CASE 1.—Mr. ——; aged 36 years; tall and spare, of regular habits; states that he has had gonorrhœa four times; first attack at the age of 18 years, and that each renewal was with stubborn gleet, and for the last six years he has had an unremitting mucoid discharge from the urethra. He placed himself under the care of several physicians of eminence, and notwithstanding patient perseverance under treatment for a considerable length of time, the annoying blennorrhœa continued unaltered.

May 2nd, 1868. He has congenital phymosis, urethral canal only admitting a No. 4 bougie, which reveals a stricture two inches from the orifice, and also at the bulb. The whole urethral canal in a granular condition. No particular spot evincing pain in external prepuce.

Introduced a No. 2 bougie, coated with mucilage, thickly coveied with powdered nitrate of silver; allowed it to crystalize, leaving two inches of the point of the bougie free to avoid unnecessary irritation of the bladder; introduced with care to secure proper contact of cantorants in the prostalic portions of the urethra; allowed it to remain ten minutes, when the nitrate of silver had all disappeared; acute pain always followed, with a frequent desire to micturate, the urine being tinged with blood. The pain, which was most intense, as the glans-penis gradually subsided in the course of a few hours. No rigor or other untoward symptom followed. A free purulent discharge was established on the following day, and expotea-tion of the mucous membrane occurred on the fifth day after the application of the escharotic. He had slight aching pains in the region of the bladder extending down the thighs, with thick mucoid deposits in his urine, which had alkaloid reaction. This constitution of his urine he remarked, had existed many months before he came under my notice. Strange to say, that after a few days treatment with diluted phosphoric acid and infusion of *Buchu*, the mucoid deposit entirely disappeared.

A fortnight after the curved bougie had been introduced, I performed Ricord's operation for phymosis, with the slight modification of uniting the mucous membrane and skin of the upper half-circle with two sutures, leaving the lower portion free to granulate, and avoid unnecessary contraction at the frænum. This healed very neatly in three weeks, leaving the glans penis quite uncovered, without any unseemly side-flaps. Two weeks more sufficed to cure the intractable blennorrhœa.

Remarks. — Specific inflammation of a mucous membrane, the result of contagion, is always considered a much more serious matter than simple inflammation resulting from an accident. In gonorrhœa, one of the specific class, the mucous lining of the urethra, in the early stage, is of a deep red color; the epithelium having disappeared, it has lost its smooth and shining appearance; and this, in its turn, is followed, as the disease advances, with erosion, ulceration, and granulation, which gradually extends back towards the prostate gland, which seems to be justly considered the seat of blennorrhœa.

A granulated condition of a canal, such as the urethra or cervix uteri, necessarily requires direct application of suitable cauterants for their healthy restoration. It is simply futile to suppose that medicine can ever affect a cure in such cases, without proper local trertment. A granular condition of the urethra may very properly be compared with the same condition of the eyelids, which are speedily brought under control with gentle cauterization.

We therefore feel warranted in urging urethral cauterization, as a safe and speedy remedy in all cases of obstinate chronic gleet.

